# The mediation role of psychological capital between family relationship and antenatal depressive symptoms among women with advanced maternal age: a cross sectional study

**DOI:** 10.1186/s12884-022-04811-y

**Published:** 2022-06-14

**Authors:** Kai Zeng, Yang Li, Rumei Yang

**Affiliations:** 1 School of Nursing, Southern Medical University, Baiyun District, Shatai South Road, Guangzhou, No.1023-1063 Guangdong China; 2 University of Texas at Austin School of Nursing, #3.446; 1710 Red River St, Austin, TX 78712 USA; 3 School of Nursing, Nanjing Medical University, No.818, Tianyun Road, Jiangning District, Nanjing, Jiangsu China

**Keywords:** Mental health, Pregnancy, Depression, Resilience

## Abstract

**Background:**

Due to changes in family policy in China, pregnancy at advanced age (30 years old or above) is prevalent. Advanced maternal age is known to be related to a variety of negative health outcomes, including antenatal depression. Family relationship quality might be an important factor for antenatal depressive symptoms among Chinese women with advanced maternal age. However, the underlying mechanisms in which family relationship quality can affect antenatal depressive symptoms among this population and how positive psychological capital (PsyCap) intervenes in this impact are not clear.

**Objectives:**

To describe the prevalence and demographic characteristics of antenatal depressive symptoms among Chinese pregnant women with advanced maternal age, and to explore the mediation effect of PsyCap in the associations between family relationship quality and antenatal depressive symptoms.

**Methods:**

We conducted a cross-sectional survey at a tertiary hospital in China. A total of 192 women with maternal age of 30 years or older completed the questionnaires. Data on antenatal depressive symptoms, PsyCap, family relationship quality and demographic characteristics were collected. The multiple mediation models in SPSS’s PROCESS macro were used to test whether PsyCap mediated the relationship between family relationship quality and antenatal depressive symptoms.

**Results:**

Approximately 28.6% of participants had antenatal depressive symptoms and 6.8% reported poor family relationship quality. Participants with higher education (*p* = .02) and better family relationship quality (*p* = .00) were less likely to have antenatal depressive symptoms. PsyCap collectively (*β* = 1.14, *p* < .05), or more specifically resilience (*β* = 0.61, *p* < .05) significantly mediated the relationship between poor family relationship quality and antenatal depressive symptoms.

**Discussion:**

The relationship between family relationship quality and antenatal depressive symptoms can be mediated by PsyCap collectively or via resilience specifically. It is important to screen antenatal depressive symptoms among pregnant women with advanced age, especially those who have poor family relationship quality. Counseling and psychotherapy initiatives for resilience-enhancing training for pregnant women at advanced age may provide a promising target to break the link between poor family relationship quality and antenatal depressive symptoms.

## Introduction

Antenatal depression is an important health concern for childbearing women. Antenatal depression is associated with obstetric complications for pregnant women [1], such as preterm birth [2], and caregiving stress and burden for the entire family [3]. It is estimated that 10%-15% of pregnant women are affected by antenatal depression, and the prevalence rate may be even higher in Asian populations (15%-20%) [4]. Studies also indicate that the risk for antenatal depression increases among women aged 30 years and older [5, 6]. Due to the transition from the “one-child policy” to “two-child policy” in China in 2015, the average maternal age of the first pregnancy increased from 26.6 in 2010 to 27.0 in 2017 [7], and pregnancy at advanced age (30 years old or above) in Chinese women is common and continues to rise [8]. However, studies focusing on antenatal depression among Chinese women, particularly those with advanced maternal age are limited [9].

In addition to the risk of advanced maternal age, Chinese women often face unique cultural challenges of family relationship quality during the childbearing years [10, 11]. Family relationship quality plays an important role in mental health for Chinese women [10]. It not only affects how a woman views her pregnancy experiences, but also affects how her partner and family feel about her childbearing [10]. In Chinese collectivistic culture, family members are quite interconnected [12], and emotionally involved with each other [13]. More importantly, family members, particularly a woman’s mother and mother-in-law have the moral responsibility to care for pregnant women [10]. Family support is the main source of assistance for Chinese women during the life transition of childbearing [14, 15]. A harmonious family relationship is a necessary precondition for women receiving family support, and thus affects their childbearing experiences and mental health [16].

Due to the significant stigmatization of mental health in Asian culture [17], many Chinese women with antenatal depression do not seek help, and thus under-recognition and under-treatment of antenatal depression among Chinese women is common [18]. To address this problem, a shift from traditional views that emphasize weakness, illness, and pathology towards a positive psychology perspective could provide a promising approach to preventing antenatal depression [19]. Unlike traditional views, positive psychology (eg. positive psychological capital [PsyCap]) stresses the inner strengths, health, and vitality of the individual [20]. PsyCap is defined as a positive psychological state or individual’s psychological capacity to cope with adversity [21]. It is characterized by four important components of self-efficacy, hope, optimism, and resilience. Self-efficacy is a positive belief in one’s ability to deal with challenging tasks; hope is defined as a positive motivational state that directs an individual towards goals and pathways; resilience refers to one’s capacity to “bounce back” from adversity to attain success; optimism refers to an explanatory style regarding self-attribution for positive events [22].

PsyCap has been extensively studied in job performance and job satisfaction, and is consistently documented as a powerful predictor or mediator of job performance and job satisfaction [23, 24]. Recently, PsyCap has been increasingly studied in the area of relationship, education, and physical and psychological health [25, 26]. A few researchers have extended PsyCap work to a focus on mental health in Chinese populations. For example, Cui et al. [27] found that PsyCap was negatively associated with depression among Chinese patients with cancer. Similarly, Cao and colleagues [28] found PsyCap served as a mediator of mental health in young adolescents. Despite the promising benefits of PsyCap, it has not been studied in a Chinese population of pregnant women. Antenatal depression is linked to obstetric complications for pregnant women [1] that imposes caregiving stress and burden on the entire family [1, 3], and Chinese women are less likely to seek help when having psychological problems [18]. Therefore, it is important to include this population in studies of antenatal depression, and to evaluate the impact of PsyCap on the association between family relationship quality and risk for antenatal depression. Based on previous studies and the following assumptions, we proposed that PsyCap functions as an important mediator. Women with higher self-efficacy may have more confidence to cope with risk during the pregnancy [29]; hope may contribute to a woman’s positive expectancy toward the fetus and their own health and have less focus on negative effects related to older age [30]; women with optimistic views may have positive attributions to pregnancy outcomes and deflect responsibility for negative events through an optimistic explanation [31]; when facing stressors during pregnancy, women with resilience may bounce back to normal life more quickly [19, 32].

**Fig. 1:**
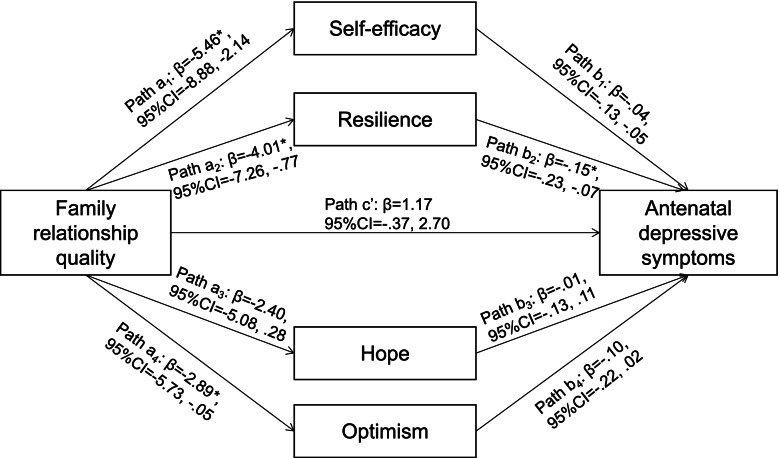
Self-efficacy, resilience, hope, and optimism as mediators of participants’ perceptions of family relationship quality on antenatal depressive symptoms. Age, living arrangement, educational background, monthly income, gestation weeks, and pregnancy related complications were adjusted in the model. CI = confidence intervals. ^*^p < .05

The present study aimed to 1) assess the prevalence and demographic characteristics of antenatal depressive symptoms among Chinese pregnant women with advanced maternal age; and 2) explore the mediation effect of PsyCap in the relationship between poor family relationship quality and antenatal depressive symptoms. Previous research has demonstrated that overall PsyCap and each component of PsyCap play important roles in mental health [20], therefore, we hypothesized that PsyCap collectively and individually functions as important mediators in the relationship between family relationship quality and antenatal depressive symptoms among Chinese pregnant women with advanced maternal age (see Fig. 1). Specifically, PsyCap might serve as a positive buffer against poor family relationship, and thus supports lower risk of antenatal depressive symptoms. By better understanding the underlying mechanisms in which family relationship quality can affect pregnant women’s mental health and how PsyCap intervenes in this negative impact, new preventive and intervention measures may be developed.

## Methods

### Design and participants

A cross-sectional study with a convenience sampling was conducted in a tertiary hospital Guangzhou, China. Participants from obstetrics and gynecology outpatient clinic for perinatal care in this hospital were surveyed between July and December 2015. Pregnant women are suggested to attend 7–11 prenatal care visits in China (an initial visit at 6–13 weeks, the second visit at 14–19 weeks, the 3^rd^ to the 5^th^ visit at 20–32 weeks, the 6^th^ visit at 33–36 weeks, and weekly until 41 weeks). The inclusion criteria were: 1) women who were pregnant at 6 weeks or more; 2) women who were at maternal age of 30 years or older; 3) women who could read and understand the survey questions. Those who had mental disorders including depression before pregnancy were excluded from this study. Pregnant women who were willing to be involved in this survey completed the questionnaires with standard guide from research assistants. Participants were promised to get feedback of assessment results in their following clinic appointments and were suggested to visit a psychological clinic if their score of Edinburgh Postnatal Depression Scale (EPDS) was 9 or above. G-Power 3.1 was used to calculate the sample size setting α = 0.05, β = 0.95, effect size = 0.15 [33], and number of predictors = 11, and then 178 participants were needed for our study. Actually, a total of 200 questionnaires were distributed by our research assistants, and 192 were returned, yielding a response rate of 96.0% (8 participants were excluded for missing demographic characteristics information).

The study was conducted in accordance with the Declaration of Helsinki and received ethical review and approval from the Institutional Review Board of Southern Medical University which the recruitment hospital was affiliated to. All surveys were anonymous and personal information other than demographics was not documented in the study. Oral informed consent was given by all participants.

### Measurements

#### Antenatal depressive symptoms

A Chinese version of Edinburgh Postnatal Depression Scale (EPDS-C) with 10 items was used to measure the levels of antenatal depressive symptoms in Chinese pregnant women. Each item was scored from 0 to 3 on a 4-point Likert scale and the total score ranged from 0 to 30. EPDS-C has been validated in the Chinese population with good reliability and validity [34]. The Cronbach’s *a* in the current study was 0.72. According to Zhao’s et al. research [35], the diagnostic cut-off point in the Chinese population was 8/9 for depression with best sensitivity (71.6%) and specificity (87.6%). Therefore, scores ≥ 9 indicated antenatal depressive symptoms in this study.

#### Psychological capital (PsyCap)

A 26-item Positive Psychological Capital Questionnaire (PPQ) was used to measure PsyCap. This scale was developed by Zhang and colleagues [36]. An exemplary item was "I can look for solutions calmly when I encounter difficulties". It is reported to have good validity and reliability in the Chinese population. PPQ consisted of four components including self-efficacy (7 items), resilience (7 items), hope (6 items), and optimism (6 items). Each item was scored on a 7-point Likert scale from 1 *(strongly disagree*) to 7 (*strongly agree*) with higher scores indicating greater positive psychological capacity. In the current study, the Cronbach’s α was 0.92 for the whole scale, 0.84 for self-efficacy, 0.73 for resilience, 0.77 for hope, and 0.83 for optimism, respectively.

#### Family relationship quality

The concept of family relationship quality was operationalized as respondents' self-perceived quality of relationship with family members including husband or partner, and/or parents or parents-in law. It was assessed by asking respondents “How would you rate your relationship with your family members who provide most care or support to you during the time you were pregnant?” on a 3-point scale from 0 = “poor” to 2 = “very good” with higher scores indicating more satisfactory relationship. The score was further dichotomized as poor or good, and coded as 0 or 1.

#### Demographic characteristics

Demographic characteristics included age, marital status, living arrangement, educational background, monthly income, gestation weeks, and pregnancy related complications.

### Data analysis

All analyses were performed using SPSS version 22.0 (SPSS Inc, Chicago, IL, USA) and SPSS’s PROCESS macro (Hayes, 2013). Continuous variables were described as mean and standard deviation, and categorical variables as frequency and percentage. Differences in continuous variables between different demographic groups were analyzed using independent sample *t*-test. A series of multiple mediation models were used to simultaneously test whether the total indirect effect exists, and if so, to what extent specific components of PsyCap mediated relationship between family relationship quality and antenatal depressive symptoms. With reference to Fig. 1, *the specific indirect effect* was defined as the product of path a and path b pertaining to a specific mediator (e.g. a_1_*b_1_ for self-efficacy), *the total indirect effect* was the sum of all the specific indirect effects (a_1_*b_1_ + a_2_*b_2_ + a_3_*b_3_ + a_4_*b_4_), and *the total effect* was the sum of the total indirect effect and direct effect (direct effect was depicted as path c’ in Fig. 1) (Preacher & Hayes, 2008). All these effects were estimated by requesting 1000 bootstrapped samples, and the statistical significance was obtained if 95% confidence intervals (CI) did not contain zero. Demographic characteristics included age, living arrangement, educational background, monthly income, gestation weeks, and pregnancy related complications known to be related to mediator and/or outcome were adjusted for in the analyses [37, 38]. Marital status was not adjusted due to its limited distribution in our sample. Statistical significance was set at *p* < 0.05.

## Results

### Prevalence and demographic characteristics of antenatal depressive symptoms

Table 1 presents descriptive information of study variables. The mean age of participants was 35.42 years (*SD* = 2.84) and 63.5% had a maternal age of 35 years and older. The majority of participants did not live with parents or parents in law (60.9%), and had a bachelor's or higher degree (56.3%). The mean score of antenatal depressive symptoms was 6.91 (*SD* = 2.96, range = 0–17, Cronbach's *α* = 0.72) with 28.6% (*n* = 55) of the participants had antenatal depressive symptoms (Table 1). Participants with higher education were less likely to have antenatal depressive symptoms than those with lower education (*p* = 0.02).

**Table 1:** Prevalence and characteristics of antenatal depressive symptoms (*N* = 192)

				Antenatal depressive symptoms	
Variables	*M* (*SD*)	Range	*n* (*%*)	*t*	*p*
Age (years)	35.42 (2.84)	30–43		1.33	.19
34 or younger			70 (36.5%)		
35 or older			122 (63.5%)		
Marital status				-	-
Unmarried or divorced			1 (0.5%)		
Married			191 (99.5%)		
Living arrangement					
Not living with parents or parents in law			117 (60.9%)	.45	.51
Living with parents or parents in law			75 (39.1%)		
Educational background				2.41	.02
Diploma or lower			84 (43.7%)		
Bachelor's or higher			108 (56.3%)		
Monthly income				1.74	.08
< $730(¥5000)			73 (38.0%)		
≥ $730(¥5000)			119 (62.0%)		
Gestation weeks					
< 28			22 (11.5%)	.43	.67
≥ 28			170 (88.5%)		
Pregnancy related complications					
None			139 (82.4%)	1.24	.22
≥ 1			53 (27.6%)		
Family relationship quality					
Poor			179 (93.2%)	2.99	.00
Good			13 (6.8%)		
Antenatal depressive symptoms	6.91(2.96)	0–17			
EPDS-C score < 9			141(71.4%)		
EPDS-C score ≥ 9			55 (28.6%)		

### Family relationship quality, psycap, and associations with antenatal depressive symptoms

As shown in Table 1, approximately 6.8% (*n* = 13) of participants reported poor family relationship quality. Participants with family relationship quality were less likely to have antenatal depressive symptoms than those with poor family relationship quality (*p* = 0.003). The mean score of PsyCap was 131.79 (*SD* = 17.83, range = 95–178, Cronbach's *α* = 0.92) for the entire scale, 35.28 (*SD* = 6.00, range = 19–49, Cronbach's *α* = 0.84) for self-efficacy, 31.70 (*SD* = 5.74, range = 14–48, Cronbach's *α* = 0.73) for resilience, 32.51 (*SD* = 4.74, range = 22–42, Cronbach's *α* = 0.77) for hope, and 32.30 (*SD* = 5.04, range = 18–42 Cronbach's *α* = 0.83) for optimism, respectively. PsyCap and its components were inversely associated with antenatal depressive symptoms, ranging from -0.32 to -0.48 (Table 2).

**Table 2:** Means, standard deviations, correlation coefficients, and Cronbach *α* coefficients for mediators and outcome variables (*N* = 192)

	*M*	*SD*	1	2	3	4	5	6
1. PsyC total scores	131.79	17.83	(*α* = .92)					
2. Self-efficacy	35.28	6.00	.86*	(*α* = .84)				
3. Resilience	31.70	5.74	.77*	.50*	(*α* = .73)			
4. Hope	32.51	4.74	.82*	.65*	.43*	(*α* = .77)		
5. Optimism	32.30	5.04	.88*	.66*	.57*	.71*	(*α* = .83)	
6. Antenatal depressive symptoms	6.91	2.96	-.48*	-.39*	-.47*	-.32*	-.41*	(*α* = .72)

The Cronbach *α* coefficients are on the diagonal. ^*^p < .05

### Test of mediation effects

The mediation effects are summarized in Table 3 and Fig. 1. The total indirect effect through self-efficacy, resilience, hope, and optimism were statistically significant in model 1 (*β* = 1.33, *p* < 0.05). This effect was retained even after controlling for covariates of age, living arrangement, educational background, monthly income, gestation weeks, and pregnancy related complications in model 2 (*β* = 1.14, *p* < 0.05), suggesting a significant overall mediation effect, i.e. family relationship affected antenatal depressive symptoms indirectly through self-efficacy, resilience, hope, and optimism collectively (Table 3). Controlling for the presence of other mediators in the model, resilience was the only significant mediator (*β* = 0.62, *p* < 0.05), indicating resilience had the greatest impact on family relationship and antenatal depressive symptoms among Chinese women at an advanced maternal age. The direct effect of family relationship quality on antenatal depressive symptoms remained non-significant after controlling for all mediators (*β* = 1.17, *p* = 0.135) (Fig. 1).

**Table 3:** Mediation effects of self efficacy, resilience, hope, and optimism in the relationship between family relationship quality and antenatal depressive symptoms

	Model 1		Model 2	
	*β*	95% CI	*β*	95% CI
Indirect effect via self-efficacy (a_1_*b_1_)	.34	-.29, 1.19	.22	.-.36, 1.02
Indirect effect via resilience (a_2_*b_2_)	.73^*^	.17, 1.50	.61^*^	.10, 1.48
Indirect effect via hope (a_3_*b_3_)	-.00	-.42, .42	.02	-.33, .49
Indirect effect via optimism (a_4_*b_4_)	.26	-.11, .93	.28	-.01, 1.03
Total indirect effect (a_1_*b_1_ + a_2_*b_2_ + a_3_*b_3_ + a_4_*b_4_)	1.33^*^	.56, 2.24	1.14^*^	.32, 1.94
Total effect (a_1_*b_1_ + a_2_*b_2_ + a_3_*b_3_ + a_4_*b_4_ + c’)	2.49^*^	.85, 4.13	2.30^*^	.64, 3.97

Age, living arrangement, educational background, monthly income, gestation weeks, and pregnancy related complications were adjusted in Model 2. *CI* Confidence intervals. ^*^p < .05

## Discussion

Advanced maternal age is an important public health concern in China. This is the first known study focusing on a better understanding of the PsyCap mechanisms on antenatal depressive symptoms in women with advanced maternal age. Nearly 28.6% of participants in the current study were affected by antenatal depressive symptoms. It appears that the prevalence rate of antenatal depressive symptoms in our study sample (30–43 years) was not higher than that in the general population of pregnant women (about 25%-28.5%) [39, 40]. Given significant stigma and underreporting of antenatal depressive symptoms in Chinese culture, we conjecture the prevalence rate of antenatal depressive symptoms might be even higher than the current finding. In addition, previous studies report mixed results on the prevalence rate of antenatal depressive symptoms among different age groups. Some studies report higher rates of antenatal depressive symptoms in pregnant women with older age [5, 41] while others observed an opposite pattern that older age was associated with lower rates of antenatal depressive symptoms [42, 43]. The discrepancy might be related to the characteristics of samples across different studies. We also found pregnant women with higher education were less likely to develop antenatal depressive symptoms while other factors including age, income, living arrangement, gestation weeks and pregnancy complications were not related to antenatal depressive symptoms. It suggests that more attention should be paid to the assessment and prevention of antenatal depressive symptoms among pregnant women with lower education at their prenatal care visits. Given a small number of the participants in our study, a larger population-based study is needed to estimate the prevalence rate of antenatal depressive symptoms in Chinese women with advanced maternal age.

Univariate analysis found family relationship quality is an important risk factor for antenatal depressive symptoms among women with advanced maternal age. This finding is consistent with other studies [44, 45], although undertaken in a general population of pregnant women. For example, Senturk et al. [44] conducted a survey on 772 pregnant women of all ages (age range = 18–44) in Turkey and found that poor family relationship quality was associated with a significant increase in antenatal depressive symptoms. Although these studies might not be completely comparable to our study due to variations in the study design, measurement, samples, and culture, we conjecture that pregnant women at advanced age and having poor family relationship quality might obtain insufficient emotional, practical and financial support from their families. This is based on the assumption that women at an older age might be better able to care for themselves than younger women whose family members might provide less support to them. In addition, strained and conflicted family relationship might also contribute to increased vulnerability, unhappiness, loneliness, helplessness, uncertainty and fatigue [12, 46]. The negative affect can further increase the risk for antenatal depressive symptoms. Family relationship quality, family support, and pregnancy experiences should be taken into account for preventing antenatal depressive symptoms in prenatal care for women at advanced maternal age.

More importantly, we found that family relationship quality may influence antenatal depressive symptoms via PsyCap overall, and more specifically via resilience. PsyCap is a metaconcept that combines various positive resources to represent psychological capacities, which are important in mitigating the impact of poor family relationship quality on antenatal depressive symptoms. This finding supports our hypothesis and is consistent with PsyCap theory [47], indicating collective contributions of all components to mental health. Although PysCap as a whole is important, resilience is even more prominent in mitigating adverse outcome. Although existing evidence pertaining to family relationship quality, resilience, and antenatal depressive symptoms is lacking, studies on general topics of resilience suggest that resilience is associated with a variety of desired health outcomes. Li and colleagues [48] found a significant indirect effect of social support on geriatric depression through the resilience in Chinese Singaporean residents aged 65 years or older. Some studies indicate that resilience has a buffering effect between major stress and depression or psychological health status among patients [49, 50]. In pregnant women, it has been found that resilience mediates the effect of adverse childhood experiences on the development of prenatal depression [51]. All of these findings indicate that resilience can help individuals to cope with negative life experiences and contribute uniquely to the desired health outcomes.

It has been proposed from previous study findings that the four components of PsyCap need to be utilized as a collective construct rather than in isolation [47]. However, these components may contribute differently to personal strengths. Researchers generally agree that resilience is related to positive psychological resource capacities including optimism, hope, and self-efficacy [52, 53]. Individuals with optimistic viewpoints, high self-efficacy, and hope are more likely to find solutions to problems, rather than ruminating on the problems and past mistakes or errors [32]. However, this does not mean that the four factors are equally important in managing stress and preventing depression. Luthans and colleagues [32] suggest that resilience is the key factor intervening in the pathway between self-efficacy, hope, and optimism, and health outcomes. In other words, these factors might not have an impact on outcomes without resilience. The possible explanation is that hope and optimism might only apply to situations involving identifiable causes and prepared action plans. In contrast, resilience can go beyond situations with planning and preparation, but also apply to situations with changes and uncertainties [47]. These findings suggest resilience might be a more robust mediator than other components and can help explain why resilience is the only significant mediator in the current study. Thus, psychological consultation and therapy initiatives such as resilience-enhancing training should be provided to pregnant women at advanced age who have risk of antenatal depression. In our study, we only addressed the collective and unique contributions of PsyCap to mental health. Future studies need to explore whether these components may interact with one another to influence an individual’s ability to cope with adversity.

## Implications

Findings from this study provide some practical implications. Firstly, health care providers need to be aware of the high prevalence of antenatal depressive symptoms in a population of Chinese women, and to establish an effective antenatal depressive symptoms assessment system to support early treatment. Secondly, it is important to assess pregnant women’s perceptions of family relationship quality during their routine clinic visits. Health care providers should not only provide information about antenatal depressive symptoms to pregnant women but also to their family members. Finally, PsyCap collectively, and resilience specifically, is an important intervention target for antenatal depressive symptoms. A synthesis of current evidence on resilience might provide insights into the future care of pregnant women and research studies that aim to strengthen pregnant women’s resilience skills.

### Strengths and limitations

Our study has several strengths. First, the sample size is enough for testing the mediation effect. Second, our study provides aids in understanding the underlying mechanisms in which family relationship quality can affect pregnant women’s mental health and how PsyCap intervenes in this negative impact. This study also has several limitations. First, the participants were only recruited from one hospital. The representation of the sample was limited and some of the results may not be generalized to other populations. Second, a single-item measure of family relationship quality might lack sufficient psychometric properties, including reliability. Low reliability could attenuate relationships among variables, making it more challenging to find significant results. However, it is easy and quick to administer as a screening tool in clinical settings. In addition, although EPDS-C is widely used, the Cronbach's *α* in the current study was 0.72, which might further reduce the statistical power. Furthermore, self-reported family relationship quality is subjective and might be intertwined with depressive symptoms such that individuals who are depressed are more likely to report poor family relationship quality. We cannot rule out the possibilities of attributional bias and recall bias due to the nature of study design. Third, causality cannot be drawn from the cross-sectional design.

## Conclusion

In this study population of Chinese women with advanced age, more than one quarter of participants were affected by antenatal depressive symptoms. PsyCap as a whole, and specifically for resilience, serves as an intervening factor to mitigate the impact of poor family relationship quality on antenatal depressive symptoms.

## Data Availability

The datasets analyzed in the current study are not publicly available due permission form the participants were not attained but are available from the corresponding author on reasonable request.

## Abbreviations

EPDS-C: Chinese version of Edinburgh Postnatal Depression Scale
PsyCap: Positive psychological capital

## References

[1] Eastwood J, Ogbo FA, Hendry A, Noble J, Page A (2017). Early Years Research Group (EYRG). The impact of antenatal depression on perinatal outcomes in Australian women. PLoS One..

[2] Miller ES, Saade GR, Simhan HN (2021). Trajectories of antenatal depression and adverse pregnancy outcomes. Am J Obstet Gynecol.

[3] Adeponle A, Groleau D, Kola L, Kirmayer LJ, Gureje O (2017). Perinatal depression in Nigeria: perspectives of women, family caregivers and health care providers. Int J Ment Health Syst.

[4] Schatz DB, Hsiao MC, Liu CY (2012). Antenatal depression in East Asia: a review of the literature. Psychiatry Investig.

[5] Ayele TA, Azale T, Alemu K, Abdissa Z, Mulat H, Fekadu A (2016). Prevalence and associated Factors of antenatal depression among women attending antenatal care dervice at Gondar University Hospital, Northwest Ethiopia. PLoS ONE.

[6] Nilsen AB, Waldenström U, Hjelmstedt A, Rasmussen S, Schytt E (2012). Characteristics of women who are pregnant with their first baby at an advanced age. Acta Obstet Gynecol Scand.

[7] Cao J, Xu W, Liu Y (2022). Trends in maternal age and the relationship between advanced age and adverse pregnancy outcomes: a population-based register study in Wuhan China 2010–2017. Public Health.

[8] Qin C, Mi C, Xia A (2017). A first look at the effects of long inter-pregnancy interval and advanced maternal age on perinatal outcomes: a retrospective cohort study. Birth.

[9] Lampinen R, Vehviläinen-Julkunen K, Kankkunen P (2009). A review of pregnancy in women over 35 years of age. Open Nurs J.

[10] Lau Y, Yin L, Wang Y (2011). Antenatal depressive symptomatology, family conflict and social support among Chengdu Chinese women. Matern Child Health J.

[11] Zhang L, Yang X, Zhao J (2020). Prevalence of prenatal depression among pregnant women and the importance of resilience: A multi-site questionnaire-based survey in mainland China. Front Psychol.

[12] Chen CH, Wang SY, Chung UL, Tseng YF, Chou FH (2006). Being reborn: the recovery process of postpartum depression in Taiwanese women. J Adv Nurs.

[13] Brathwaite AC, Williams CC (2004). Childbirth experiences of professional Chinese Canadian women. J Obstet Gynecol Neonatal Nurs.

[14] Hu Y, Wang Y, Wen S (2019). Association between social and family support and antenatal depression: a hospital-based study in Chengdu, China. BMC Pregnancy Childbirth.

[15] Yin X, Sun N, Jiang N (2021). Prevalence and associated factors of antenatal depression: systematic reviews and meta-analyses. Clin Psychol Rev.

[16] Atif M, Halaki M, Raynes-Greenow C, Chow CM (2021). Perinatal depression in Pakistan: a systematic review and meta-analysis. Birth.

[17] Woo BK, Mehta P (2017). Examining the differences in the stigma of dementia and diabetes among Chinese Americans. Geriatr Gerontol Int.

[18] Goodman SH, Dimidjian S (2012). The developmental psychopathology of perinatal depression: implications for psychosocial treatment development and delivery in pregnancy. Can J Psychiatry.

[19] Ma R, Yang F, Zhang L (2021). Resilience mediates the effect of self-efficacy on symptoms of prenatal anxiety among pregnant women: a nationwide smartphone cross-sectional study in China. BMC Pregnancy Childbirth.

[20] Seligman ME, Csikszentmihalyi M (2000). Positive psychology. an introduction. American Psychologist.

[21] Luthans F, Youssef CM (2004). Human, social, and now positive psychological capital management: Investing in people for competitive advantage. Organ Dyn.

[22] Luthans F, Avolio BJ, Avey JB, Norman SM (2007). Positive psychological capital: measurement and relationship with performance and satisfaction. Pers Psychol.

[23] Bitmiş MG, Ergeneli A (2013). The role of psychological capital and trust in individual performance and job satisfaction relationship: a test of multiple mediation model. Procedia Soc Behav Sci.

[24] Gupta V, Singh S (2014). Psychological capital as a mediator of the relationship between leadership and creative performance behaviors: empirical evidence from the Indian R&D sector. Int J Hum Resour Manag.

[25] Luthans F, Youssef-Morgan CM (2017). Psychological capital: an evidence-based positive approach. Ann Rev Org Psychol Org Behav.

[26] Broad JD, Luthans F (2020). Positive resources for psychiatry in the fourth industrial revolution: building patient and family focused psychological capital (PsyCap). Int Rev Psychiatry.

[27] Cui CY, Wang Y, Zhang Y, Chen S, Jiang N, Wang L (2021). The development and validation of the psychological capital questionnaire for patients with cancer the psychological capital questionnaire. BMC Cancer.

[28] Cao S, Zhu Y, Li P, Zhang W, Ding C, Yang D (2022). Age difference in roles of perceived social support and psychological capital on mental health during COVID-19. Front Psychol.

[29] Mo PKH, Fong VWI, Song B, Di J, Wang Q, Wang L (2021). Association of perceived threat, negative emotions, and self-efficacy with mental health and personal protective behavior among Chinese pregnant women during the COVID-19 pandemic: cross-sectional survey study. J Med Internet Res.

[30] Delale EA, Novokmet N, Fuchs N (2021). Stress, locus of control, hope and depression as determinants of quality of life of pregnant women: Croatian Islands’ Birth Cohort Study (CRIBS). Health Care Women Int.

[31] Hobbs C, Vozarova P, Sabharwal A, Shah P, Button K (2022). Is depression associated with reduced optimistic belief updating?. R Soc Open Sci.

[32] Luthans F, Vogelgesang GR, Lester PB (2006). Developing the psychological capital of resiliency. Hum Resour Dev Rev.

[33] Kang H (2021). Sample size determination and power analysis using the G*Power software. J Educ Eval Health Prof.

[34] Guo X, Wang Y, Liu Y, Chen J, Pu XF (2009). Study on the optimal critical value of the Edinburgh Postnatal Depression Scale in the screening of antenatal depression. Chin J Nurs.

[35] Zhao Y, Kane I, Wang J, Shen B, Luo J, Shi S (2015). Combined use of the postpartum depression screening scale (PDSS) and Edinburgh postnatal depression scale (EPDS) to identify antenatal depression among Chinese pregnant women with obstetric complications. Psychiatry Res.

[36] Zhang K, Zhang S, Dong Y (2010). Positive psychological capital: Measurement and relationship with mental health. Stud Psychol Behav.

[37] Babalola SS (2009). Women entrepreneurial innovative behaviour: the role of psychological capital. Int J Bus Manage.

[38] Zhao Y, Kane I, Mao L (2016). The prevalence of antenatal depression and its related factors in Chinese pregnant women who present with obstetrical complications. Arch Psychiatr Nurs.

[39] Zeng Y, Cui Y, Li J (2015). Prevalence and predictors of antenatal depressive symptoms among Chinese women in their third trimester: a cross-sectional survey. BMC Psychiatry.

[40] Zhou C, Zheng W, Yuan Q (2018). Associations between social capital and maternal depression: results from a follow-up study in China. BMC Pregnancy Childbirth.

[41] Carlson DL (2011). Explaining the curvilinear relationship between age at first birth and depression among women. Soc Sci Med.

[42] Qiao YX, Wang J, Li J, Ablat A (2009). The prevalence and related risk factors of anxiety and depression symptoms among Chinese pregnant women in Shanghai. Aust N Z J Obstet Gynaecol.

[43] McMahon CA, Boivin J, Gibson FL (2011). Age at first birth, mode of conception and psychological wellbeing in pregnancy: findings from the parental age and transition to parenthood Australia (PATPA) study. Hum Reprod.

[44] Senturk V, Abas M, Berksun O, Stewart R (2011). Social support and antenatal depression in extended and nuclear family environments in Turkey: a cross-sectional survey. BMC Psychiatry.

[45] Elif Y (2017). Adolescent or advanced age pregnancy: what about quality of life?. Eurasian J. Med Oncol.

[46] Heshmati S, Blackard MB, Beckmann B, Chipidza W (2021). Family relationships and adolescent loneliness: An application of social network analysis in family studies. J Fam Psychol.

[47] Youssef CM, Luthans F (2007). Positive organizational behavior in the workplace: the impact of hope, optimism, and resilience. J Manag.

[48] Li J, Theng YL, Foo S (2015). Does psychological resilience mediate the impact of social support on geriatric depression? an exploratory study among Chinese older adults in Singapore. Asian J Psychiatr.

[49] Sharpley CF, Bitsika V, Wootten AC, Christie DR (2014). Does resilience ‘buffer’ against depression in prostate cancer patients? a multi-site replication study. Eur J Cancer Care (Engl).

[50] Liu JC, Chang LY, Wu SY, Tsai PS (2015). Resilience mediates the relationship between depression and psychological health status in patients with heart failure: a cross-sectional study. Int J Nurs Stud.

[51] Howell KH, Miller-Graff LE, Schaefer LM, Scrafford KE (2020). Relational resilience as a potential mediator between adverse childhood experiences and prenatal depression. J Health Psychol.

[52] Kotera Y, Green P, Sheffield D. Positive Psychology for Mental Wellbeing of UK Therapeutic Students: Relationships with Engagement, Motivation, Resilience and Self-Compassion. Int J Ment Health Addict. 2021;1–16. 10.1007/s11469-020-00466-y. 10.1007/s11469-020-00466-y PMC7802612 33456408

[53] Kotera Y, Cockerill V, Chircop J, Kaluzeviciute G, Dyson S (2021). Predicting self-compassion in UK nursing students: Relationships with resilience, engagement, motivation, and mental wellbeing. Nurse Educ Pract.

